# Granulomatosis With Polyangiitis With Multifocal Pancreatic and Renal Pseudotumors, Mimicking Neoplasm: A Case Report

**DOI:** 10.1155/crra/6883674

**Published:** 2026-08-25

**Authors:** Surbhi Singh, Akhil Tanwar, Dhrumil Patel, Jonathan Von Reusner, Prabath Kumar Mondel, Ashlesha Udare

**Affiliations:** ^1^ Department of Abdominal Radiology, Thomas Jefferson University Hospital, Philadelphia, Pennsylvania, USA, jefferson.edu; ^2^ Department of Pathology, Thomas Jefferson University Hospital, Philadelphia, Pennsylvania, USA, jefferson.edu; ^3^ Department of Neuroradiology, Thomas Jefferson University Hospital, Philadelphia, Pennsylvania, USA, jefferson.edu

**Keywords:** abdominal granulomas, granulomatous polyangiitis, inflammatory pancreatic mass, inflammatory pseudotumor, multiorgan

## Abstract

Granulomatosis with polyangiitis (GPA) is an ANCA‐associated small‐vessel vasculitis that can rarely present as a mass‐like inflammatory pseudotumor in the abdomen. We report a 52‐year‐old woman with systemic symptoms and laboratory evidence of GPA with multiple heterogeneous mass lesions involving the pancreas and both kidneys on CT imaging. These lesions mimicked a neoplasm, prompting further characterization with tissue sampling. Histopathology of the pancreatic lesion confirmed granulomatous inflammatory changes. The patient was treated with glucocorticoids and rituximab, resulting in marked progressive resolution of lesions on follow‐up imaging. This case highlights the importance of recognizing atypical mass‐like abdominal manifestations of GPA, the pivotal role of cross‐sectional imaging in diagnosis and follow‐up, and the need to consider inflammatory pseudotumor in the differential diagnosis to avoid unnecessary surgery.

## 1. Introduction

Granulomatosis with polyangiitis (GPA) is a systemic autoimmune disorder classified among the antineutrophil cytoplasmic antibody (ANCA)–associated vasculitis (AAV) that typically affects small‐ to medium‐sized blood vessels, leading to widespread inflammation. Mass‐like inflammatory lesions or pseudotumors are uncommon manifestations of GPA and are often called granulomas since histological examination shows granulomatous inflammation and rarely vasculitis [1]. This case highlights the rare mass‐like pancreatic and renal involvement and discusses the role of imaging in diagnosis and follow‐up.

## 2. Case Report

**Figure 1:**
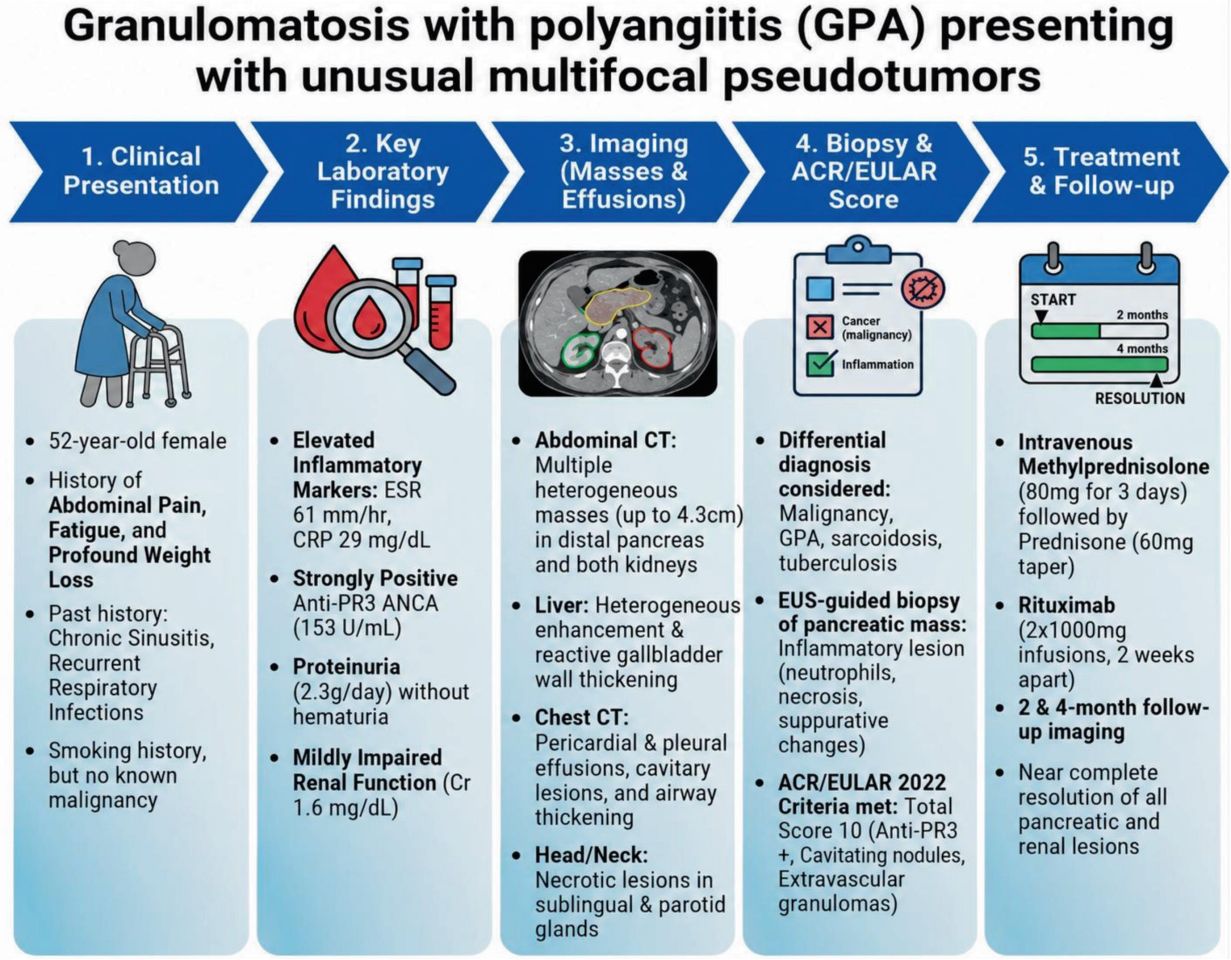
Timeline of clinical presentation, diagnostic test, and treatment.

**Figure 2:**
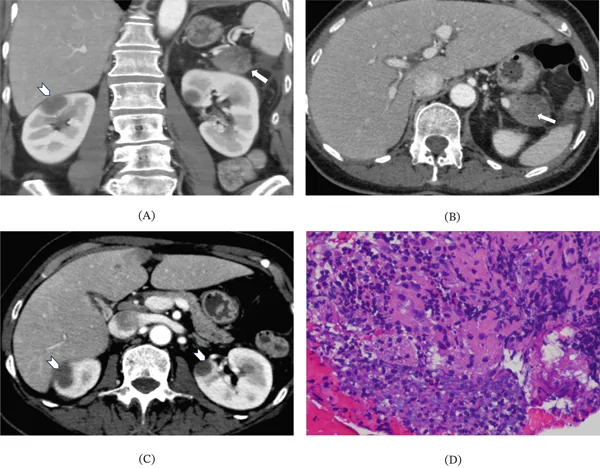
CT abdomen and pathology at initial presentation. (A,C) CT showed several hypoenhancing solid mass lesions in the bilateral kidneys representing inflammatory masses (arrowheads). (B) A heterogeneous enhancing mass lesion is seen in the tail of the pancreas with hypodense internal necrotic areas, mimicking a pancreatic tumor. (D) Histopathology of the pancreatic lesion shows areas of suppurative inflammation with neutrophils and necrosis.

**Figure 3:**
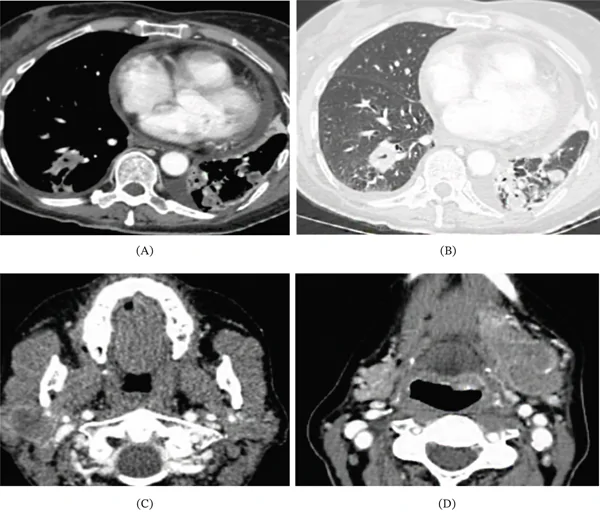
Extra‐abdominal findings: CT chest shows (A) pericardial and mild pleural effusions, (B) with multiple scattered nodules with central cavitation in bilateral lungs, representing granulomatous lesions. Contrast‐enhanced CT neck shows hypodense infiltrative lesions in (C) the right parotid and (D) the left sublingual gland.

A 52‐year‐old female presented with a history of abdominal pain, weight loss, and fatigue. He had a past history of chronic sinusitis and recurrent respiratory infections. Laboratory findings showed elevated inflammatory markers (ESR was 61 mm/h and CRP was 29 mg/dL) and elevated anti‐PR3 ANCA levels of 153 obtained by ELISA (Inova Quanta Lite; reference range: 0–20: normal, 21–30: weak positive, and > 31: strong positive). The urine analysis reveals proteinuria of 2.3 g/day without hematuria. Renal function was mildly impaired, with a serum creatinine of 1.6 mg/dL (Figure 1). The patient had a history of smoking, but there was no known history of malignancy. A contrast‐enhanced CT of the abdomen and pelvis was performed on a 256‐slice Philips scanner, which showed multiple heterogeneous hypodense masses (largest measuring 4.3 cm) involving the distal body/tail of the pancreas and both kidneys (Figure 2, CT collage with four figure parts). These lesions had irregular margins appearing infiltrative with a heterogeneous enhancement pattern on portal venous phase images acquired at 70 s postcontrast injection. The liver showed heterogeneous enhancement with reactive gallbladder wall thickening. Pericardial and pleural effusions were seen at the lung bases, and additional cavitary lesions and airway thickening were seen on dedicated chest CT (Figure 3A,B). Head and neck imaging showed necrotic‐appearing lesions in the sublingual and parotid glands (Figure 3C,D) with normal brain MR. Given the multifocal appearance of these lesions, a differential diagnosis of inflammatory pseudotumors of GPA, sarcoidosis, and tuberculosis was considered. Carbohydrate antigen 19‐9 and carcinoembryonic antigen were within normal limits. However, given the profound weight loss and unusual‐appearing pancreatic masses, EUS‐guided sampling of the pancreatic lesion was performed, which confirmed inflammatory lesions showing neutrophils, necrosis, and suppurative changes (Figure 2D). The case met diagnostic criteria according to ACR/EULAR 2022 revised classification criteria for GPA, with a total score of 10, including anti‐PR3 ANCA positive (+5), cavitating pulmonary nodules (+2), and extravascular granulomatous inflammation on biopsy (+3).

**Figure 4:**
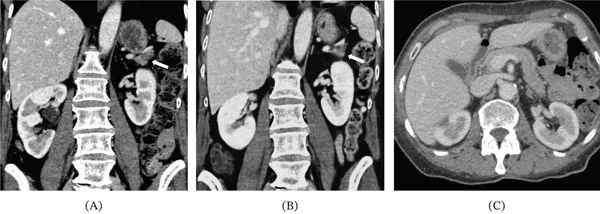
Follow‐up imaging at (A) 2 months and (B,C) 4 months after the completion of steroid treatment. CT abdomen shows progressive decrease in the size of the pancreatic tail lesion (arrows) and near‐complete resolution of renal lesions.

The patient was started on intravenous methylprednisolone 80 mg for 3 days, followed by prednisone 60 mg and immunosuppression with rituximab, with two 1000 mg infusions given 2 weeks apart. Follow‐up studies were done at 2‐ and 4‐month intervals after starting treatment, which showed a progressive decrease in the size of pancreatic and renal lesions, with near‐complete resolution in the most recent imaging (Figure 4).

All patient data and imaging are anonymized for protection of patients′ privacy. A formal review of the case report was done by the institutional privacy office, which certified the case to be completely deidentified. This case report follows the CARE guidelines for case reports and includes all aspects of a case report. This case report follows the CARE guidelines for case reports and includes all aspects of its checklist.

## 3. Discussion

The 2012 International Chapel Hill Consensus Conference revised nomenclature to divide granulomatous vasculitis into large‐vessel (giant cell arteritis and Takayasu arteritis), medium‐vessel (mainly polyarteritis nodosa), and small‐vessel AAV (GPA), which appear similar on pathology. Clinical manifestations are heterogeneous and can be classified as granulomatous (which are associated with increased risk of relapse) or vasculitis (which are associated with increased mortality). The diagnosis of GPA relies on a combination of clinical findings, imaging study results, laboratory test results such as eosinophil count, serologic markers (ANCAs directed against proteinase 3 are present in approximately 65%–75% of individuals with GPA), and histopathologic results. Early immunosuppression with glucocorticoids and rituximab or cyclophosphamide is essential; surgery should be avoided unless malignancy cannot be excluded or complications arise [2, 3]. Prognosis depends on timely therapy and the extent of organ involvement, with relapses common and long‐term monitoring required [4]. Virtually any organ can be involved, and cross‐sectional imaging, particularly contrast‐enhanced CT, has a crucial role in the diagnosis and follow‐up of patients with abdominal involvement in GPA [5].

Abdominal manifestations of GPA other than glomerulonephritis are rare, and the small intestine is the most common site of involvement [6]. GPA can rarely present with pancreatic and renal masses that mimic malignancy, leading to diagnostic delay and inappropriate surgery. Patients with solitary renal masses associated with GPA are hypovascular on imaging and may exhibit a favorable response to steroid or immunosuppressive treatment [7, 8]. Pancreatic involvement in GPA is rare and usually presents as acute or recurrent pancreatitis [9–11] or pancreatic exocrine insufficiency [12], less commonly as a mass [13]. Acute pancreatitis has been described as the initial presentation of GPA in a few case reports, which had a rapidly progressing course with severe multiorgan involvement [14, 15]. Less than a dozen cases of pancreatic mass‐like involvement have been described in the literature, most of which were retrospectively diagnosed after surgical resection [16–18]. Although biopsy may suffice when there are other supporting GPA criteria, surgery may be the only resort, particularly if the ANCA is negative [18]. Differentials also include other granulomatous diseases like sarcoidosis and tuberculosis. Similar imaging characteristics, including heterogeneous enhancing granulomatous lesions with cavitation, can be seen commonly in the lungs and lymph nodes in all these conditions; however, remarkable response to steroids, serum markers, and histopathology can help with differentiation. The literature shows multiple cases of identical appearance of GPA lesions and tuberculosis, especially in the chest and nasopharyngeal locations; however, involvement of abdominal organs like kidneys and pancreas is rare in TB or sarcoid [19].

Our case is unique, as it has a multiorgan GPA pseudotumor involving both kidneys and the pancreas, which resolved with treatment.

### 3.1. Teaching Points

Granulomatous pseudotumors can have similar imaging characteristics to malignancy, and correlation with clinical and lab values is essential in differentiating them. When multifocal masses cross anatomical boundaries and involve atypical sites (e.g., salivary glands, pancreas, and kidneys simultaneously), systemic inflammatory diseases like GPA, IgG4‐related disease, sarcoidosis, and tuberculosis must be included in the differential. Tissue biopsy and positive ANCA serology are key diagnostic contributors in some uncertain cases.

## 4. Conclusion

This case underscores the importance of imaging in diagnosing and managing GPA, particularly in cases with multiorgan involvement. Early recognition and appropriate treatment are critical to prevent long‐term organ damage and improve patient outcomes. The prognosis is generally favorable with early diagnosis and treatment with immunosuppressant therapy; however, long‐term monitoring with laboratory and serological markers should be continued for detection of recurrence.

## Funding

No funding was received for this manuscript.

## Conflicts of Interest

The authors declare no conflicts of interest.

## Data Availability

Data sharing is not applicable to this article as no datasets were generated or analyzed during the current study.
